# Current News

**Published:** 1891-12

**Authors:** 


### Current News.

### MISSOURI STATE DENTAL ASSOCIATION.

THE Twenty-seventh Annual Meeting of the Missouri State Dental Association was held at Louisiana, Mo., July 7 to 10, inclusive. The following officers were elected for 1892: President, Dr. George L. Shepard, Sedalia, Mo.; Vice-President, Dr. E. E. Shattuck, Kansas City, Mo.; Second Vice-President, Dr. J. T. Fry, Moberly, Mo.; Corresponding Secretary, Dr. William Conrad, St. Louis, Mo.; Recording Secretary, Dr. W. M. Carter, Sedalia, Mo.; Treasurer, Dr. J. A. Price, Weston, Mo.

*Board of Censors.*—Dr. C. J. McBride, Perryville; Dr. W. H. Buckley, Liberty; Dr. C. Lindsley, St. Louis, Mo.

*Committee on Ethics.*—Dr. F. Slater, Rich Hill; Dr. E. B. Crane, California; Dr. E. W. Bear, Sedalia, Mo.

*Publication Committee.*—Dr. E. E. Shattuck, Kansas City; Dr. W. S. Lowry, Kansas City; Dr. W. E. Tucker, Springfield, Mo.

*Committee on Law.*—Dr. J. A. Price, Weston, Mo.

*Committee on New Appliances.*—Dr. J. B. Vernon, St. Louis.

The next meeting will be held at Clinton, Mo., the first Tuesday after July 4, 1892.

WILLIAM CONRAD,
*Corresponding Secretary.*

### MASSACHUSETTS DENTAL SOCIETY.

THE following were elected officers of the above Society at its last annual meeting, held in Boston, July 9 and 10, 1891:

President, Dr. George F. Eames, Boston. Vice-Presidents, Dr. J. W. Ball, Boston; Dr. W. E. Page, Boston. Secretary, Dr. Edgar O. Kinsman, Cambridge. Treasurer, Dr. Edward Page, Charlestown. Librarian, Dr. Joseph King Knight, Hyde Park.

*Executive Committee.*—Dr. R. R. Andrews, Dr. Joseph King Knight, Dr. W. E. Boardman, Dr. H. S. Draper, Dr. V. C. Pond.

DR. EDGAR O. KINSMAN,
*Secretary.*

15 BRATTLE SQUARE, Cambridge, Mass.

### MISSISSIPPI VALLEY MEDICAL ASSOCIATION.

The Mississippi Valley Medical Association held its Seventeenth Annual Session at St. Louis, October 14, 15, and 16, 1891, President Dr. C. H. Hughes, of St. Louis, in the chair. The attendance was large, the papers numerous and valuable. Dr. I. N. Love, the incomparable chairman of the Committee of Arrangements, and his able assistants, deserve unstinted praise for their provision of receptions, rides, dinners, suppers, banquets, fine weather, and full moon. Dr. C. A. L. Reed, of Cincinnati, was elected President; Dr. E. S. McKee, of Cincinnati, re-elected Secretary; Dr. C. S. Bond, Richmond, Ind., First Vice-President; Dr. J. H. Stucky, Louisville, Ky., Second Vice-President; Dr. Joseph Ransohoff, Cincinnati, Chairman Committee of Arrangements. Place of meeting, Cincinnati, October, 1892.

---

### INVITATION TO ANNIVERSARY MEETING OF THE FIRST DISTRICT SOCIETY OF NEW YORK.

The members of the profession are cordially invited to attend the Twenty-third Anniversary Meeting of the First District Society, to be held at the Academy of Medicine, No. 17 West Forty-third Street, New York City, January 18, 19, and 20, 1892.

This meeting will be one of prime interest, and a large attendance is confidently expected. The essays to be read will be in the hands of the committee, in advance, and copies will be supplied to those who have kindly promised to discuss the topics. About thirty eminent men will thus be upon our programme, prepared in advance, to take part in the proceedings, in addition to which there will be the usual general discussion, open to all who attend.

As heretofore announced, our clinics will be devoted to the introduction of entirely new methods, inventions, appliances, instruments, or medicaments. We have already arranged for several, which alone will repay those who attend, and are in correspondence about others, which will probably be secured. Any desiring a place in our clinics should write immediately to Dr. F. A. Roy, No. 148 West Seventieth Street, New York.

The Academy of Medicine is convenient to several fine hotels, where rooms may be secured at one dollar per day. Gentlemen who decide to attend may obtain all information, as to hotels, railroads, etc., and receive special programme of meeting, as soon as ready, by addressing the chairman of the committee.

The profession will kindly accept this as an official invitation to our meeting, and not wait for a special one.

M. L. Rhein, M.D., D.D.S.
George H. Winkler, D.D.S.
Rodrigues Ottolengui, M.D.S., *Chairman*,
115 Madison Avenue.

---

Corrosive Sublimate as a Disinfectant against the Staphylococcus Pyogenes Aureus.—A. C. Abbott (*Johns Hopkins Hospital Bulletin*, No. 12, April, 1891) publishes the results of tests made upon cultures of the staphylococcus pyogenes aureus with a 1 : 1000 solution of corrosive sublimate. Abbott finds that the disinfectant power of corrosive sublimate in the above concentration, when tested by methods which exclude the carrying over of minute quantities of the disinfectant, is not so great as has been claimed. He holds that in many of the experiments heretofore made to test corrosive sublimate the latter has been assigned a higher rank than it deserves, because some of it has been transferred to the culture medium, and has inhibited, but not destroyed, the growth.

His experiments were made upon liquid cultures containing sterilized sand. With cultures of this sort he was able, by filtration, to get a better distribution of the organisms in the liquid, avoiding macroscopic clumps, which might interfere with the action of the disinfectant upon the organisms in the centre. Suspensions in water were also used, also filtered. Fresh cultures and fresh solutions of corrosive sublimate were used, of course, in every case.

Abbott finds that the number of organisms makes a difference in the efficacy of the disinfectant. The greater the number of organisms the more difficult the disinfection. Cultures vary in their resisting power,—organisms from one culture resisting better than those from another. Cultures in beef-tea resist better than suspensions in water. Organisms which remain alive after the action of the disinfectant are retarded in their growth and are weakened in virulence. Corrosive sublimate, in the proportion of 1 : 400,000, retarded growth in cultures of bouillon containing peptone; 1 : 600,000 without peptone. The staphylococci, which have been attenuated by the action of the sublimate, regain their virulence when cultivated for some time on ordinary culture media. —B. Meade Bolton, M.D., Haagland Laboratory, Brooklyn.

TURPENTINE AS A GERMICIDE AND ANTISEPTIC.—Although the oil of turpentine (*oleum terebinthinæ*, U.S.P.) is not unknown as an antiseptic and germicide, its insolubility in water and irritating properties have hitherto made its use impracticable. That it has its special uses, however, in this connection, I have had abundant testimony.

It is a well-known fact among naturalists that, if the air of a cabinet be impregnated with the vapor of turpentine, the specimens are safe from the ravages of moths and like intruders so long as this condition of the air of the cabinet remains.

Having learned the advantage of turpentine in preserving entomological specimens, I concluded to try its germicidal properties in cases containing surgical instruments. A bacteriological examination made four weeks afterwards, and compared with the examination of cases not provided with turpentine, convinced me of its efficacy, and I soon afterwards applied the same principle to drawers containing towels, gauze, bandages, etc.

The method is simple. The turpentine is placed in flat, large-mouthed bottles at the bottom of each case or drawer, the volatility of the turpentine causing the vapor to impregnate the surrounding air.

Of late I have also placed my surgical instruments, the night preceding an operation, in a flat dish containing oil of turpentine. The instruments are completely sterilized, are not injured by the submersion, and are easily dried by a piece of sterilized gauze or towel. The characteristic odor of turpentine can be removed by ether.

The cheapness of turpentine, and the ease with which it may always be obtained, added to its special adaptability in preserving the aseptic condition of instruments, bandages, etc., by its vapor, may make it a valuable addition to the list of antiseptics and germicides.

I have also used benzol in the above manner. Its greater volatility gives it a more rapid germicidal action than turpentine, but its great inflammability admonishes caution in its use.—W. SCHLEPPEGREL, A.M., M.D., New Orleans, in *Medical News*, May 30, 1891.

---

MICROCIDIN—A NEW ANTISEPTIC.—M. Polaillon (*Journal de Médecine de Paris*) presented to the Académie, in the name of Dr. Berlioz, professor at the medical school of Grenoble, an experimental and clinical study of microcidin. He thinks that antiseptic therapeusis is not more generally employed by the public because of the inconvenience of the antiseptics now in use, such as the mercuric salts, phenol, naphthol, thymol, salicylic acid, boric acid, etc., which are dangerous on account of their toxicity or caustic properties, or of disagreeable odor, or uncertain or inconvenient to handle because of their insufficient solubility in water. The remedy in question possesses a very feeble toxicity, is caustic, and is very soluble in water, at the same time being without odor or taste. It is a combination of naphthol and soda. One part of microcidin is soluble in three parts of water. In medicinal doses, even the largest required, it is not caustic; it is unirritating when applied to wounds, is odorless, and may be used for toilet purposes. It does not corrode instruments or dressing materials. It reduces fever rapidly, and is excreted by the urine, which is rendered aseptic. It has been employed successfully in a large number of cases of infected and operative wounds. It is used in aqueous solution in the strength of 5 to 1000 (strong solution) and 3 to 1000.—*L'Organe de la Confraternité Médicale*, July, 1891, p. 163.

---

PYOKTANIN is a failure as an antiseptic, says Dr. Roswell Park (*Annals of Surgery*). It cannot be relied upon in surgery except in strength that is dangerous. As an injection in gonorrhœa, he adds: "I have had no experience with it, but find that most of those who have tried it have met with disappointment. Upon granulating surfaces it does not appear to be stimulating and to exert a desirable effect, but no more so than other substances within easy or easier reach, and its stain is often undesirable. In ophthalmological practice it appears also to have scarcely come up to the requirements of the day. On the whole, then, it has but few qualities by which we are to commend it above numerous other drugs of its general class, while in all that may answer to the more scrupulous demands of aseptic surgery it has proved in my hands—as in those of others who have tested it from the purely clinical stand-point—disappointing."—*Medical Record*.

---

DERMATOL.—By this name Dr. R. Heintz, of Breslau, designates a new chemical preparation, the basic gallate of bismuth, which he recommends as an antiseptic with varied therapeutic uses, that may be used advantageously in the place of iodoform. Dermatol is a fine, absolutely inodorous powder, of saffron-yellow color, non-hygroscopic, and unaffected by light and air. As it is insoluble in ordinary vehicles, it can only be employed in powder. Besides its strong antiseptic properties, it has a stimulating and astringent action which makes it valuable in the treatment of wounds and ulcers, in that it hastens cicatrization. On account of its insolubility, poisoning by it is impossible; it does not produce even a local irritation. According to Dr. Heintz, dermatol may be substituted for iodoform in all cases where the latter is indicated. It may also be used internally, in daily doses of two grammes (thirty grains), in diseases of the digestive tract, especially in the profuse diarrhœa which is present in catarrhal and ulcerous affections of the intestines.—*Revue Médico-Pharmaceutique*, June 30, 1891, p. 111.

---

LOCAL ANÆSTHETIC FOR COMPARATIVELY PAINLESS EXTRACTION OF TEETH.—As one of the great corps of "country doctors" who act in almost every capacity for their patrons, I submit the following formula as a local anæsthetic for the almost painless extraction of teeth:

℞ Hydrochlorate cocaine, 5 parts;
Crystal. carbolic acid, 6 parts;
Pine gum camphor, 6 parts;
Ninety-five per cent. alcohol, q.s. to make 120 parts. M.

Inject one to three minims of this mixture, with a hypodermic syringe, deeply into the gum on the inner and outer sides of the tooth. Apply over the gum a piece of absorbent cotton wet in the solution. Wait four to five minutes. The gum can then be freely incised and the tooth drawn with a minimum amount of pain. Try it.

J. WILTON HOPE, M.D.

POQUOSIN, YORK CO., VA.

—*Virginia Medical Monthly.*

---

DR. J. E. SHOEMAKER (*Journal of the American Medical Association*) gives some vigorous thrusts at those who oppose antiseptic methods. We append abstracts:

"*When is Antisepsis a Failure?*—Outside of a comparatively small circle of surgeons there are heard from time to time suggestions, which occasionally appear in print, that the system of 'Listerism,' so-called, is a failure. Strange as it may seem, it is not very uncommon to hear some one say that in a given case 'every antiseptic precaution' was adopted, but the result was bad. The speaker would have you believe that he had done his part and that the system was at fault.

"Now, it is worth while to consider briefly where the difficulty lies; and without entering the discussion of asepsis as opposed to antisepsis,—absence of dirt *versus* sterilization of dirt,—without advocating special methods or dressings, attention may be drawn to practical difficulties which lead to misunderstanding.

"A few men, like Mr. Tait, vigorously attack the theory of Listerism, while they themselves carry out the principles underlying its success. The reputation of Mr. Tait, however, rests upon his operative work, and not upon his opinions or his explanations. When, in characteristic style, he says (*British Medical Journal*, September 27, 1890, p. 287), . . . 'The tone and attitude adopted by Sir Joseph Lister, at Berlin, clearly show that the whole sad business is on its last legs,' etc.; also (p. 729): 'I venture to say that before the present generation has run out the word "antiseptic" will be all that is left to represent this strange structure,' the harm that he can do is not great among the men who are doing the best work in surgery, especially in general surgery. These cannot work for a day without discovering for themselves that their results are better or worse, according to their greater or less microscopic and chemical cleanliness in operating. Active surgeons do not care how Mr. Tait explains his good results. He might refuse to believe in the law of gravitation if he choose, but as long as he did not violate it, as long as he refrained from walking out of windows and off precipices, his opinion as to the law would make little difference. Most men care little that he denies the evil potency of germs and relies upon removing decomposable material from his wounds. They remember that he deals with a peculiar membrane and its neighborhood, that he is extremely clean in his work, and they will permit him to attack Sir Joseph Lister personally, and his impregnable principles to his heart's content,—principles of the widest practical application. The harm Mr. Tait can do is to unsettle the mind of the man who is beginning his work; and, worse than that, his writings tend to salve the conscience of those who have had no training in genuine aseptic methods, who fail consequently to fully carry them out, and who joyfully hail any champion who even seems to justify their indifference.

"But even among the better-trained class of men does not one often see a lamentable failure to grasp the essential ideas of surgical cleanliness?

"There are hundreds of men to-day who apparently persuade themselves that mopping a 1 to 20 carbolic acid, or a 1 to 2000 bichloride of mercury solution about a wound area constitutes using 'every antiseptic precaution,' as the phrase goes. There are also men who will use chemicals upon a septic patient, but neglect to change infected bedding. There are men who will go to an operation with the points of their scissors, the locks and serrations of their hæmostatic forceps, the eyes of their needles, choked with dried blood or worse material from the last operation. They never boil an instrument. Their conscience is satisfied with the carbolic acid in the instrument pan. Some men wash their hands before an operation no better than before dinner. When an instrument or a sponge drops to the floor they may rapidly rinse it in the pan and use it at once. There are other men who have trained nurses, sterilized dressings, and boiled instruments, but who, after they have washed for the operation, shake hands with a spectator, put a hand in a pocket, remove instruments from an old blood-stained case, help carry a table, handle dusty bottles, or use a handkerchief, and yet say they use every antiseptic precaution. Many men know better. What is lacking is careful self-training and what may be called an aseptic conscience. What is wanted is a realizing sense of the real difficulty in getting things clean and then keeping them so. Carbolic acid solutions as practical sterilizers are a delusion and a snare. They work slowly at best. Unless too strong for comfortable or safe handling they do little good, and they do enormous harm by quieting the conscience of the man who ought to spend more time in cleansing his hands; yet how many times do we see them relied upon when they only cover dirt. Antisepsis is a failure when it is superficial. . . .

"There is no doubt that the great principle of cleanliness in surgery, whether obtained by soap, hot water, dry heat, or chemicals, has come to stay, and the sooner all of us act thoroughly upon that principle, ignoring personal discussion, the better."

